# DownLink amplified loss function module for 5G air to terrestrial communication networks

**DOI:** 10.1371/journal.pone.0332303

**Published:** 2025-09-17

**Authors:** Ammar Armghan, Sultan S. Aldkeelalah, Monia Hamdi, Somia Asklany, Chahira Lhioui, Rim Hamdaoui, Paolo Mercorelli, Ali Elrashidi

**Affiliations:** 1 Department of Electrical Engineering. College of Engineering, Jouf University, Sakaka, Saudi Arabia; 2 Department of Information Technology, College of Computer and Information Sciences, Princess Nourah bint Abdulrahman University, Riyadh, Saudi Arabia; 3 Department of Computers and Information Technologies, College of Sciences and Arts Turaif, Northern Border University, Arar, Saudi Arabia; 4 Department of Computer Science and Artificial lntelligence, College of Computing and Information Technology, University of Bisha, Bisha, Saudi Arabia; 5 Department of Computer Science, College of Science and Human Studies-Dawadmi, Shaqra University, Shaqra, Saudi Arabia; 6 Institute for Production Technology and Systems (IPTS), Leuphana Universität Lüneburg, Lüneburg, Germany; 7 Electrical Engineering Department, College of Engineering, University of Business and Technology, Jeddah, Saudi Arabia; Beijing Institute of Technology, CHINA

## Abstract

Combining air-to-ground network experiences with Fifth Generation (5G) communications, inferred path loss and Doppler impacts on communication dependability due to the difference in downlink sampling rates of receiver amplification gain intensities. This work addresses the challenges of route loss and Doppler effects in dynamic communication scenarios by integrating air-to-ground communication concepts with 5G networks. This research combines air-to-ground communication concepts with 5G networks to improve signal dependability, compensating for path loss and Doppler effects. The introduced Down-Link Amplified Loss Function Module (DALFM) dynamically adjusts amplification and sampling procedures to offset transmission losses. The module optimizes signal gain with a recurrent learning architecture to provide dependable communication under various conditions. Experimental validation attests that DALFM enhances transmission gain by 8.12%, sample rate by 10.67%, and reduces path loss by 9.57%. The findings bring their capacity to enhance 5G air-to-terrestrial networks into perspective, paving the way for more solid and flexible wireless communication.

## 1. Introduction

### 1.1. Background

Amplification becomes the determining factor for expanding communication in air-to-ground 5G networks, with environmental signal attenuation and distance being the primary issues [1,2]. Signal power during transit must always be maintained to ensure the integrity of communication. Adaptive amplifiers counteract signal degradation by minimizing loss and compensating for speed variation and distance through quality enhancement [3,4]. Compensation is necessary to overcome structural and atmospheric variability, providing reliable communication between airborne and ground nodes [5,6]. Detection and reduction of path loss are primary challenges for terrestrial communication systems, particularly due to the increasing data transmission rates and the growing distance between communication points [7]. Path loss is defined as the weakening of signal strength as it propagates through a medium. It is primarily determined by the conditions provided by the medium through which it travels, such as terrain, obstacles, and atmospheric variations [8]. Detecting an accurate path loss level is crucial for establishing communication systems with high-quality, robust features that enable seamless data transfer [9]. Several measures have been suggested to mitigate path loss, and among them, advanced antenna designs, beamforming, and signal processing techniques will be heavily relied upon to reduce signal degradation due to distance or hostile environments [10]. In an integrated satellite-terrestrial network, lower path loss is also essential, as it helps preserve high data rates across vast areas with minimal signal loss. Integrating smart resource allocation and frequency management techniques helps optimize the transmission process, ensuring the signal remains strong across different nodes in communication [11,12].

Machine learning (ML) applications in terrestrial communications are being increasingly applied to mitigate path loss effects by detecting and compensating for signal degradation [13]. Advanced algorithms, such as reinforcement learning and deep neural networks, offer real-time prediction and adjustment of transmission parameters. This is used to optimize signal strength at the point of communication and reduce losses [14,15]. Given the environmental and operational factors, such algorithms have been trained to recognize the pattern of signal degradation, allowing them to dynamically scale their transmission power, frequency, and routing [16]. More complicated scenarios of signal environments are being developed through ML models like convolutional neural networks and recurrent neural networks [17]. Adaptive models adapt as they gather more data, whereas the detection and compensation of path loss improve with more samples. These models are, therefore, highly viable in dynamic environments, such as integrated satellite-terrestrial networks. Another aspect is that the use of ML techniques will not only increase the efficiency of signal transmission but also reduce the need for manual intervention when dealing with path loss [18,19]. Signal quality, error correction, and interference in down-link conditions are all improved by including an amplified loss function within the module. Particularly in high-demand, dynamic network environments, DALFM demonstrates measurable improvements in accuracy and efficiency compared to standard neural networks. Nevertheless, more optimization is required for real-time applications due to the higher computing complexity introduced by this invention. Overall, DALFM is a step closer to solving problems unique to communication; it will pave the way for next-generation networks to utilize neural networks, which are more resilient and adaptable.

Dynamic 5G air-to-terrestrial (A-t-T) communication introduces Doppler shifts due to the relative velocity between transmitting and receiving nodes, such as UAVs, satellites, and terrestrial base stations. Synchronization errors, inter-carrier interference (ICI) in OFDM-based systems, and Doppler shifts, which alter the signal frequency, lead to signal degradation in high-mobility scenarios. These impacts complicate communication, unlike route loss, which depends on distance and environment. The recommended DALFM indirectly reduces Doppler degradations by optimizing sample rates and using adaptive amplification. Recurrent learning adjusts for transmission distortions to improve downlink communication and minimize errors. This limits the frequency shifts of the received signal. This module’s adaptive frequency correction, which does not directly counter Doppler changes, helps maintain reliable communication in high-mobility situations. Another example is channel-dependent real-time amplification changes. The main contributions of the paper are:

1. A down-link amplified loss function module is developed to improve the communication rate of air-to-terrestrial 5G networks through precise sampling and amplification over different intervals.
2. A recurrent learning model governs the selection and detection processes to estimate the transmission gain, error, and path loss in varying downlink allocated intervals.
3. The proposed module’s assessment utilizes dedicated metrics, including transmission gain, error, and path loss, as well as hyperparameter analysis related to the loss function and learning decisions.

### 1.2. Integration with existing 5G protocols and implications for 6G networks

DALFM can be efficiently integrated into 5G network slices using edge computing and 5G NR support. DALFM can dynamically allocate resources based on offered QoS requirements in network slicing, with real-time amplification and adjustment of sampling rates. Running DALFM on edge computing environments, such as MEC servers, offloads computational workloads from central cores, providing enhanced responsiveness in high-mobility situations. Moreover, its compatibility with 5G NR protocols ensures a seamless transition, utilizing current modulation schemes such as OFDM and beamforming, thereby improving downlink stability without necessitating fundamental infrastructure changes. DALFM for 6G coordinates with AI-driven network optimization to achieve autonomous adaptation in dynamic environmental changes. DALFM can combat significant propagation loss over THz and mmWave frequencies, ensuring proper data transfer with high-frequency networks. DALFM can also accommodate integration in space-air-ground-based communication architectures, providing improved signal dependability in changing environments. Further research will focus on developing enhanced real-time learning schemes and scalability for next-generation wireless networks.

The remainder of the paper is organized as follows: Section 2 presents a literature review on the proposed topic. Section 3 provides a detailed description of the proposed Down-Link Amplified Loss Function Module. Section 4 gives the results and discussion, while the paper’s conclusion is finally drawn in Section 5.

## 2. Related works

Current research has explored various methods to further enhance air-to-terrestrial (A-t-T) communication networks by reducing path loss, optimizing resource allocation, and improving signal reliability.

### 2.1. Machine learning for path loss mitigation

Nauman et al. [13] also suggested a multi-agent reinforcement learning approach for resource allocation in integrated terrestrial-satellite networks, dynamically adjusting network parameters to enhance signal quality. Birabwa et al. [20] proposed the User Association technique known as multi-agent dueling double deep Q network (MA3DQN). Decision-making is decentralized to maximize data rate and minimize handoffs. The method applies the concept of MA3DQN, which enables users to make self-driving decisions on network association, thereby increasing data rate with minimal handoffs. The method also provides efficient resource allocation within a dynamic environment, significantly contributing to overall network efficiency. Yin et al. [21] proposed a resource allocation model for Satellite-Terrestrial Integrated Networks (STINs). The common drawback of resource allocation schemes is that these methodologies cannot make a practical tradeoff between power consumption and quality of service. This method jointly optimizes routing, bandwidth, and power allocation to ensure power usage efficiency in satellite power, thereby meeting user demands efficiently. The strategy enhances the network’s overall sustainability and considerably improves resource management.

### 2.2. Integrated satellite-terrestrial networks for reliable communication

Kwon et al. [22] proposed the Integrated Terrestrial-Satellite Networks method. The integration is significant as it could substantially offset interference and difficult propagation conditions that have hampered performance. The approach involves in-band access-backhaul transmission combined with reverse time division duplexing, which minimizes interference and offers more efficient, interference-free communication across networks. Cooperation between base stations is necessary to establish more reliability in dynamic environments. Sun et al. [23] designed a coordinated multi-point (CoMP) transmission and non-orthogonal multiple access (NOMA) CoMP-NOMA Unmanned Aerial Vehicle (UAV) Network technology to enhance the unmanned aerial vehicle network. Traditional techniques fail to address the problem of coverage and ergodic rate when dealing with heterogeneous user distribution under high mobility. The approach is very suitable for dynamic user assignment and efficient resource usage. The integration has significantly improved the network’s performance, particularly when multiple aerial and terrestrial users are present. Li et al. [24] introduced the Rate-Splitting Multiple Access (RSMA) method. Adopting RSMA improved the efficiency of both broadcast and unicast channels, as well as the approaches, in terms of enhanced spectral efficiency and more efficient resource allocation. The approach optimizes the max-min rate performance of users, bringing balanced utilization between satellites and terrestrial components. The method achieves significant overall performance improvements through enhanced service delivery and efficient resource management.

### 2.3. Path loss estimation and signal optimization

Kong et al. [25] proposed the semi-grant-free (SGF) Integrated Satellite-Aerial-Terrestrial Networks to enhance the uplink transmission via these networks. The challenges associated with interference and imperfect channel conditions are the primary problems in uplink communication. These issues are addressed by semi-grant-free transmission and zero-forcing beamforming, which ensure higher performance and reliability in uplink throughput. Adaptive power allocation enhances throughput and improves reliability in the uplink, particularly in adverse channel conditions. Perihanoglu et al. [26] proposed a GeoAI-based path loss estimation method to enrich the estimation of losses in complex environments. The approach utilizes GeoAI-based models that consider spatial data to calculate path loss, particularly in heterogeneous environments accurately. Improved estimation is necessary for network planning optimization and global performance. It provides the vital information that aids in effective network management. Sun et al. [27] introduced the concept of a reconfigurable intelligent surface (RIS) in Duplex UAV Networks to enhance duplex communication performance. The system suffers from many problems with the standard approach due to interference and hardware imperfections. The new method introduces RIS, which improves signal strengths while minimizing interference power. The overall communication performance can be improved with enhanced system resilience in challenging environments. Qu et al. [28] designed wavelength shift keying (WSK) for the Free-Space Optical Networks technique to enhance the performance of free-space optical (FSO) networks. FSO networks typically experience signal degradation due to atmospheric turbulence. The method enhances receiver sensitivity by modulating the intensity, combined with wavelength shift keying (WSK). The system offers reliable data transmission without turbulence in atmospheric states, ensuring versatile FSO communications.

### 2.4. Adaptive resource allocation and network optimization

Teng et al. [29] introduced the radio frequency/millimetre wave method in non-orthogonal multiple access networks. The method conquers the shortcomings of traditional access schemes based on user distance and beamforming in hybrid radio frequency and millimetre wave communications. It improves the users’ allocation and enhances the reliability of communications based on closed-form expressions for outage probability. Zhao et al. [30] proposed the Adaptive Resource Allocation technique. The technique leverages passive reflection surfaces deployed for simultaneous message transmission between access points and sensor nodes to counter the threat of satellite-based eavesdroppers. It optimizes the adaptive power design problem to maximize energy efficiency, subject to the constraint of the eavesdropper’s maximum data rate. The optimal solution obtained demonstrates significant improvements in energy efficiency and quality of service, while ensuring security in 6G networks. Han et al. [31] proposed RSMA for integrated terrestrial networks, enabling dynamic spectrum sharing and rate-splitting multiple access in integrated satellite-terrestrial networks. The method effectively reduces interference in multiple-access environments and overcomes the limitations of the spectrum. A non-orthogonal broadcast and unicast model has been introduced, featuring four possible transmission schemes designed to maximize the minimum rate of terminals under balanced spectrum utilization. Compared to traditional methods, the method provides significantly better service delivery for broadcast and unicast communications.

### 2.5. Advancements in network integration and resource optimization for terrestrial and non-terrestrial communications

Pugliese et al. [32] analyzed the integration of Terrestrial and Non-Terrestrial networks with integrated access and backhaul technology. The approach enables the integration of non-terrestrial networks and satellite backhauling in fifth-generation systems, supporting future sixth-generation architectures. A comprehensive review examines the challenges and feasibility of integrating Low Earth Orbit (LEO) and Geostationary Earth Orbit (GEO) satellites in hybrid integrated access and backhaul architectures. The results indicate that problems in the satellite systems are the limiting factors for successful integration. Kryszkiewicz et al. [33] proposed Vehicle-to-Vehicle (V2V) Communications with TV Frequencies for Platooning. This study builds on previous work by employing similar strategies within a different communication framework to enhance signal reliability in high-mobility conditions. The 5.9 GHz band is highly congested, resulting in communication reliability issues. Utilizing terrestrial TV frequencies, the method introduced a double-slope, double-shadowing model that accurately predicts the signal behavior in these bands, thereby providing greater reliability in the communication framework. Enhanced communication among vehicles leads to an improvement in platooning reliability compared to traditional frequency bands. Zhao et al. [34] developed the Integrated Sensing and Communication (ISAC) method to enhance bandwidth management in Internet-of-Things satellite-terrestrial relay networks. The technique integrates an Integrated Sensing and Communication framework that integrates sensing and communication abilities. The framework dynamically groups users into different groups based on the results provided by wireless sensing, ensuring that the developed groups allocate an appropriate amount of bandwidth. The approach ensures optimal uplink access to Internet-of-Things devices, thereby improving system throughput and reducing packet loss rates. Yin et al. [35] proposed a joint computation offloading (JCO) and resource allocation method in space-air-terrestrial. The technique involves the introduction of a three-tier computing framework for improving the computation offloading of devices without ground communication facilities. The aim is to minimize the total execution cost while adhering to time and capacity constraints.

The major challenge is a mixed-integer non-linear programming problem, for which relaxation and the Majorize-minimise techniques were used. The DALFM adopts a distinct approach to addressing congestion. Instead of using a different frequency band, it optimizes amplification and sampling for better downlink communication. This way, it ensures robustness without changing the frequency spectrum. A recurrent learning architecture drives adaptive amplification and sampling processes in the proposed Down-Link Amplified Loss Function Module (DALFM), outperforming earlier approaches in the literature. Communication in 5G air-to-terrestrial (A-t-T) networks is optimized for enhanced performance. DALFM achieves reliability within the present spectrum by optimizing sampling and targeting amplification, unlike models such as Kryszkiewicz et al. [33], which require additional frequency bands to reduce congestion.

In contrast to machine learning methods like MA3DQN [20], which focus on user association and resource allocation, the suggested module dynamically estimates and compensates transmission losses and Doppler shifts to maintain signal quality during dynamic intervals. DALFM utilizes real-time transmission gain and path loss prediction to ensure uninterrupted communication, reducing the impact of path loss by 9.57% and increasing the sampling rate by 10.67% compared to WSK-FSO [28] and JCO-MM [35]. The robustness of mega-constellation satellite networks to extensive geographical failures, including natural disasters or regional outages, is examined by Ouyang, Ye, and An (2025) [36]. The influence of regionally correlated failures on network connectivity and service continuity is methodically modeled in this study. The authors highlight significant weaknesses in satellite placement and interlink configurations through simulation and topological analysis. Their results underscore the importance of robust design techniques in enhancing fault tolerance for future global satellite networks. Proliferated Low Earth Orbit (LEO) satellite network topologies are tested for failure resilience in military and mission-critical communication scenarios by Shake et al. (2022) [37]. The article compares the node and link failure responses of LEO constellation topologies, assessing connection, latency, and rerouting. The authors compare topological configurations using simulations to identify those with improved fault tolerance. They offer crucial insights for creating resilient LEO networks that can operate in severe environments. Table 1 summarizes the research gap in existing literature and outlines how DALFM fills such gaps in 5G air-to-terrestrial communication.

**Table 1:** Research gap in existing approaches and DALFM’s contributions.

Category	Existing Approaches & Limitations	Research Gap	DALFM’s Contribution
**Machine Learning for Path Loss Mitigation**	ML-based methods (e.g., MA3DQN, deep reinforcement learning) focus on resource allocation and routing.	Lack of real-time adaptability in adjusting amplification and sampling rates based on channel variations.	DALFM dynamically optimizes amplification and sampling using recurrent learning for real-time adjustments.
**Integrated Satellite-Terrestrial Networks**	Satellite-aided models (e.g., RSMA, CoMP-NOMA) enhance coverage but require additional infrastructure to support their operation.	Dependence on satellite networks makes them less adaptable for standalone 5G terrestrial environments.	DALFM operates within existing 5G terrestrial networks, ensuring seamless adaptation without extra infrastructure.
**Adaptive Resource Allocation**	Resource allocation techniques (e.g., JCO-MM, RF/mmWave) improve efficiency but lack signal compensation.	Existing methods fail to mitigate path loss directly and Doppler shifts in dynamic environments.	DALFM actively compensates for path loss and Doppler effects by dynamically tuning amplification levels.
**Path Loss Estimation & Signal Processing**	GeoAI-based and RIS-aided methods estimate path loss but do not integrate dynamic compensation.	Absence of real-time loss function adaptation to varying network conditions.	DALFM utilizes an amplified loss function to adjust transmission parameters, ensuring stable communication continuously.

## 3. Proposed down-link amplified loss function module

### 3.1. System model and initialization

**Fig 1:**
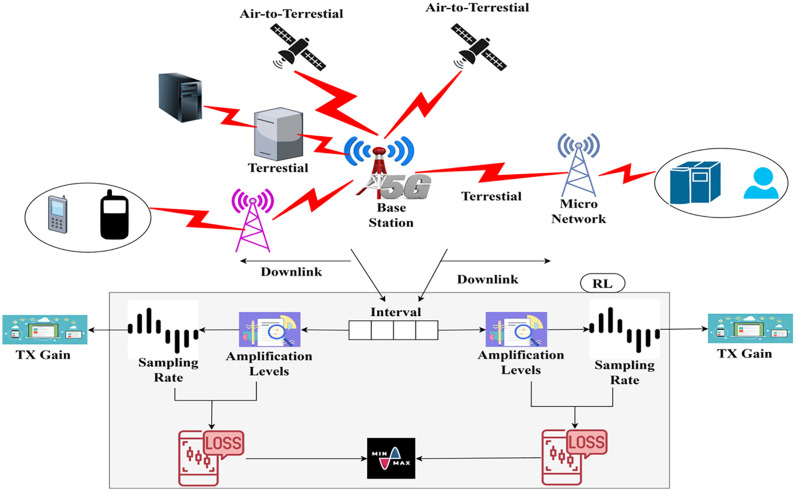
System model.

The routing protocols in the 5G generation are complex due to the high demand for devices and mobility, resulting in communication latency and delay as resources are handled. This mechanism illustrates uplink and downlink communication, where the base station receives the signal as input and performs the necessary actions for uplink and downlink communication. Here, the sampling rate is considered when the amplification levels are initiated at different intervals. This is performed to address the loss and ensure a higher transmission gain by reducing the loss rate. By executing this process, the downlink amplified mechanism is defined and addresses the loss and error caused by amplification and sampling rate variations at different time intervals. This evaluation aims to analyze the path loss, a preliminary step in this proposal. Since this work is done on air-to-terrestrial networks, the Down-Link Amplified Loss Function Module (DALFM) has been developed. The system model for the proposed function module is illustrated in Fig 1. The Down-Link Amplified Loss Function Module’s (DALFM) amplification technology is built to meet the basic standards of linear amplification, guaranteeing distortion-free and non-linear signal amplification. Critical for 5G air-to-terrestrial communication systems, this technology ensures the preservation of signal integrity by maintaining proportionality between the input and output. This linearity is well-suited to the demanding performance requirements of next-generation communication networks. It reinforces the fundamental amplification properties and enhances the network’s capacity to manage high data rates with reduced interference.

The communication pursues different frequency levels to reduce sampling errors and achieve optimal transmission gain. The downlink communication stream provides the DALFM module with its principal inputs, which are the transmit gain (TX Gain), sampling rate, and amplification level. Path loss is calculated using statistical error estimation (min/max loss detection) while these inputs are observed over predetermined transmission intervals. Updated amplification parameters and sample rates are the DALFM module’s outputs, which are then sent back into the system to optimize transmit gain and reduce signal deterioration.This work aims to improve the transmission gain and reduce the amplification loss and sampling rate. This module identifies the system gain during frequency sampling at different amplification levels. The amplification is required to prevent transmission losses between distinct intervals across various pervasive downlinks. The downlink amplified loss function module is developed in a 5G environment where air-to-terrestrial communication occurs efficiently without loss and delay. The adaptive error correction techniques and optimal loss function amplification enable DALFM to overcome the problems caused by path loss, including signal attenuation and distortion. Without changing the fundamental physical principles of path loss, this method improves the overall performance and reliability of 5G air-to-terrestrial (A-t-T) networks. By examining this mechanism, the initial step is to analyze the path loss equated in Equation (1) below.


$$A^{\prime }=\frac{\text{1}}{s_{l}}\times \sum_{q_{c}}^{s_{l}\in S_{r}}\left(A_{e}\times D_{t}\right)*{\left|s_{l}\right|}_{d_{w}}^{u_{k}}+u_{c},\;for\;all\;u_{c}\in p_{a}$$

$$q_{c}=\left(s_{l}+A_{e}\right)\times \frac{L_{s}}{\prod_{u_{c}}D_{t}}+\left\{\left[\left(d_{\text{0}}+s_{l}\right)\times \left(d_{w}-u_{k}\right)\right]-v_{n}\right\}$$

$$D_{t}=\left(d_{w}-u_{k}\right)\times \sum_{q_{c}}\left(s_{l}+A_{e}\right),\;for\;all\;D_{t}\in s_{l}$$

It is derived as,


$$p_{a}=u_{c}+\frac{\text{1}}{d_{n}}\times \sum_{s_{l}}\left(q_{c}+d_{\text{0}}\right)-v_{n}$$ (1)

The analysis for path loss is expressed in Equation (1) above, where the signal and frequencies are acquired at varying time intervals. The analysis is represented as $\begin{array}{c} \;A^{\prime } \end{array}$, and the signal frequencies are symbolized as  s the time interval is denoted as $\;v_{n}$. The sampling rate, attenuation, and weighting factor are symbolized as uplink and downlink. Here, the sampling loss is identified and labelled as $\;L_{s}$, where the path loss is labelled as $\;p_{a}$, occurs during communication, and it is represented as $\;u_{c}$. The data is $\;d_{\text{0}}$, whereas $\;d_{n}$ represents the number of data signals. From this analysis step, the signal and frequencies are determined to enhance the transmission gain, considering the sampling rate and amplification levels. The minimum and maximum rates are utilized to enhance downlink communication. The overall concept is to improve communication between devices connected via air-to-terrestrial communication modules. This category defines the loss signal that leads to error, and is taken into consideration where the path losses are detected $\;p_{a}\left(D_{t}\right)\to d_{n}-v_{n}$. The path losses are detected $\;D_{t}\left(p_{a}\right)+s_{l}-v_{n}$, it is observed on the time interval. According to the evaluation step, it estimates the sampling rate and amplification levels. By evaluating this downlink, accurate signal processing is carried out. Based on this evaluation step, the path losses are detected at different intervals, and the amplified loss function is calculated using the following Equation (2).


$$\begin{array}{c} C_{a}\left(A_{e}\right)=\prod_{d_{\text{0}}}^{d_{n}}\left(v_{n}+u_{c}\right)\times \left\{\left(s_{l}+e^{\prime }\right)q_{c}+{\left(B_{\text{0}}\times q_{c}\right)}^{-\text{1}}\right\} \end{array}$$

$$e^{\prime }\left(D_{t}\right)=B_{\text{0}}\times \sum_{S_{r}}\left(v_{n}-L_{s}\right)+\frac{\text{1}}{d_{n}},\;for\;all\;L_{s}\in u_{c},$$

It is computed as,


$$L_{s}\left(D_{t}\right)=\sum_{s_{l}\in B_{\text{0}}}^{q_{c}}\left(S_{r}*d_{\text{0}}\right)\times u_{c}+A^{\prime },\;whereas,\;u_{c}\;\forall \;v_{n}$$

$$d_{\text{0}}\left(u_{c}\right)=\frac{d_{w}+u_{k}}{\sum_{\left(F_{a}+w_{i}\right)}B_{\text{0}}+S_{r}}-v_{n}$$ (2)

The amplification loss function is calculated, and it is described as $\;C_{a}$, here the error rate is detected and labelled as $\begin{array}{c} \;e^{\prime } \end{array}$. The base station process is $\;B_{\text{0}}$, which performs the sampling for the downlink communication. This category is used to define $\;v_{n}-\left\{L_{s}-e^{\prime }\right\},\;for\;all\;v_{n}\in u_{c}$. This evaluation step is considered to find the frequency of the signal and provides an estimate of the optimality of the downlink communication $\;d_{w}\left(B_{\text{0}}\right)+q_{c}$The fading and shadowing are symbolized as $\;F_{a}\;and\;w_{i}$. Both of these parameters enhance the amplification, which is an obstacle during air-to-terrace communication. This case examines the optimal amplification levels for downlink and uplink communication. The sampling and amplification rates are considered to verify the error signal and provide communication estimation. This case illustrates that the loss function addresses the error rate and improves the transmission gain. The evaluation utilizes the signal processing loss factor to determine the path loss and enhance communication. If any communication error occurs, the losses are due to the delay, fading, and shadowing factors. This examination of the base station with the device is used to forward the signal to the terrestrial and establish communication. This step is considered and provides an optimal sampling rate for the signal processing for air-to-terrestrial 5G communication. From this evaluation step, errors and losses are identified and reduced to improve the transmission gain. The downlink communication initiated improves the transmission standard gain. Here, the examination of downlinks and their communication factors is discussed by introducing recurrent learning.

### 3.2. Amplification level and sampling validation

Recurrent learning is defined as feeding the input as the signal with the previous state of processing and analysis of the output at varying intervals, which is the current state. The maximum amplification rate required in the last interval defines the previous state, and the difference-based level for the active interval defines the current state. This holds the hidden state of action used as the training phase to improve the transmission gain observed in the downlink communication. The current and previous states are processed under the communication state, ensuring the activation function that indicates the sampling rate and amplification levels. The Equation (3) below is used to examine downlink communication.


$$M_{e}=d_{w}\times {\left\|u_{c}+d_{0}\right\|}_{p_{a}}^{L_{s}}-e^{\prime },\;for\;all\;L_{s}\in S_{r}$$ (3)

**Fig 2:**
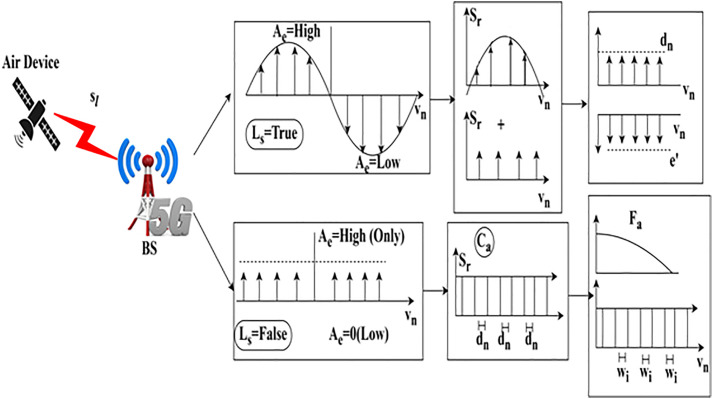
Amplification loss function with and without sampling loss.

The examination is done for downlink communication, and it is described as $\;M_{e}$, where the losses and errors are detected $\;\left(L_{s}+e^{\prime }\right)\times D_{t}$, examination is $\;M_{e}$. This step involves analyzing the sampling rate and amplification level to estimate the base station’s computation. The $\;C_{a}$ without and with loss is portrayed in Fig 2.

The sampling rate for the transmission rate during intervals is used to verify $v_{n}$ allocation under $L_{s}=false\;$ and true cases (Fig 2 reference). The $A_{e}=low$/ high is defined using $\;\left[\left(S_{r}\times d_{o}\right)+A^{\prime }\right]\forall \;v_{n}$; the $L_{s}$ is true if the case of $e^{\prime }\left(D_{t}\right)$ is true for $\;C_{a}\left(A_{e}\right)$. Therefore $F_{a}$ and $\;w_{i}$ does not suit this process, wherein the $d_{n}$ and  e′ are alone distinguished. In the $L_{s}=false\;$case $S_{r}$ is differentiated for $F_{a}$ and $w_{i}$ in any $\;v_{n}$. This requires $(\frac{e^{\prime }-L_{s}}{\left(F_{a}+W_{i}\right)}\forall \;A_{e}$=high/low cases, and thus, the sampling for new intervals relies on the $\left(e^{\prime }-L_{s}\right)$ observed in $\left(v_{n}-\text{1}\right)$ interval. Therefore $C_{a}\;\forall \left\|u_{e}+d_{o}\right\|$ and $\left(s_{l}+v_{t}\right)$ defines the amplification level required for $\;u_{c}$. The processing step indicates the $d_{w}*{\left\|u_{c}+d_{\text{0}}\right\|}_{p_{a}}^{L_{s}}$ where the path losses are considered. Path losses lead to communication losses, and it is described as considered into account, and it is described as $\;v_{t}$. The process is carried out where downlink communication is executed at varying intervals. The path loss is computed using Equation (1), where the error rate is calculated and reduced in further evaluation. The state of observing the downlink of the losses is addressed, whether it is maximum or minimum. The progression indicates the recurrent learning concept where the sampling rate. The verification of lower loss shows higher transmission gain. This execution is carried out by considering fading and shadowing, which relies on $\;F_{a}+w_{i}(A^{\prime }+e^{\prime })$. It defines the path losses and indicates the acquisition of input as the signal, and computes the lower loss rates $\;L_{S}\left(D_{t}-v_{n}\right)\times \sum_{p_{a}}u_{c}$. The signal is given as the input, which acquires the process from the previous state and is given to the network. The current state processes the input and provides the output, deciding the sampling rate under the downlink communication. After these performances, the sampling rate is identified and derived below. Mathematical formulas that seem to describe a system of activities or processes are these Equation (4). They could be related to communication models, network optimization, or resource allocation.


$$I_{d}=\frac{\text{1}}{d_{n}}+\sum_{p_{a}\in u_{c}}^{q_{c}}s_{l}+\left(d_{w}\times D_{t}\right),\;for\;all\;d_{w}\;\forall \;A_{e}$$

$$I_{d}=\frac{D_{t}}{L_{s}}\times \sum_{M_{e}\in e\prime }^{u_{c}}\left(v_{t}-u_{s}\right)\times C_{a},\;for\;all\;u_{s}\in s_{l}$$

$$I_{d}=\frac{A^{\prime }}{d_{n}}+\sum_{u_{c}\in e^{\prime }}^{p_{a}}d_{w}\times \left(v_{t}+s_{l}\right),\;for\;all\;v_{t}\in q_{c}\left(s_{l}\right)$$

$$I_{d}=\frac{C_{a}}{p_{a}}\times \sum_{q_{c}\in v_{n}}^{s_{l}}M_{e}+\left(S_{r}+A_{e}\right),\;for\;all\;S_{r},A_{e}\in d_{w}$$ (4)

**Fig 3:**
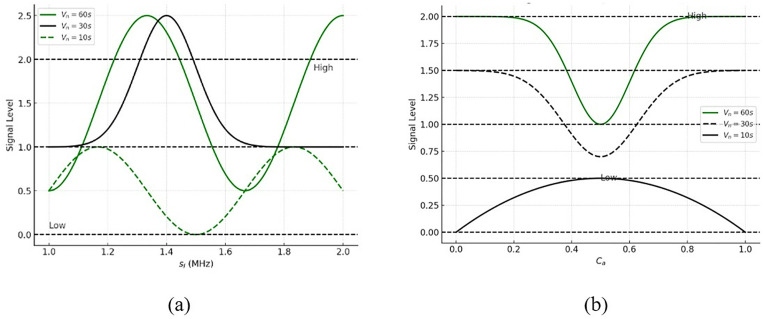
(a) High and (b) low analysis of sampling rate.

The identification is performed for the sampling rate that exhibits the n of devices, and identification is described as $\;I_{d}$. The analysis is conducted for varying sampling rates and amplification levels, ensuring reliable downlink communication. The execution of the downlink process uses a recurrent learning mechanism. This process is considered by calculating the amplification loss function that computes the $\;\left(S_{r}\times A_{e}\right)\times \left\{A^{\prime }+d_{n}\right\}$. The base station acquires the input signal and provides the training phase for the losses and error-based signals, which are extracted as the output. The recurrent learning is performed under the downlink communication. This indicates the fading and shadowing process, illustrating the analysis calculation. The $\;S_{r}$ analysis analysis for its rates (high and low) is analyzed using Fig 3.

Fig 3 illustrates the variations in the signal-to-noise ratio (SNR) over time. Through three separate communication periods, the signal loss and capacity are determined. In the first case, we correct for the distance between the communicating nodes once the product of the transmitted signal level and the amplification factor equals 1 or 0, indicating the presence of high and low outcomes, respectively. The capacity-to-distance ratio is a loss-suppressing factor that helps reduce signal deterioration by distributing communication resources across multiple intervals and considering signal levels on all paths. The transmission parameters are assigned according to the distance between the nodes to maintain an uninterrupted communication channel in the absence of signal loss. The capacity and amplification factors are fine-tuned to maintain optimal communication efficiency when the loss rate and signal amplification are in perfect balance. Under ideal conditions of transmission and amplification, this equilibrium causes the signal loss to be proportional to the signal-to-noise ratio/distance. The outcome is a rough approximation of route loss, which may be determined for different communication scenarios in the same way as explained.

### 3.3. Path loss estimation

Identifying the sampling rate reduces the occurrence of path loss in the network and ensures communication without loss or delay. Here, it defines a signal-based process that analyzes the input as a signal, considers the previous state, and provides the resultant output. In this execution stage, the identification of the sampling rate involves the number of samples taken continuously during communication to produce a discrete or digital signal. From this, the evaluation takes on a different time interval. This case computes the path of losses. Thus, the sampling for every second is captured to observe the digital signal. Hence, the sampling rate is detected, and the formulation below in Equation (5) indicates the hidden layer process.


$$H_{0}=\left\{ \begin{array}{c} I_{d}\left(v_{n}(0)-v_{t}\right)=\left(A^{\prime }+S_{r}\right)+d_{0}\times \prod_{d_{w}\in u_{c}}^{A_{e}}s_{l(0)}+\left(\frac{1}{d_{n}}*D_{t}\right)-e^{\prime }+r_{t}(0)\to u_{s}\\ I_{d}\left(v_{n}(1)-v_{t}\right)=\left(A^{\prime }+S_{r}\right)+d_{1}\times \prod_{d_{w}\in u_{c}}^{A_{e}}s_{l(1)}+\left(\frac{1}{d_{n}}*D_{t}\right)-e^{\prime }+r_{t}(1)\to u_{s}\\ \vdots \\ I_{d}\left(v_{n}(n)-v_{t}\right)=\left(A^{\prime }+S_{r}\right)+d_{n-1}\times \prod_{d_{w}\in u_{c}}^{A_{e}}s_{l(n-1)}+\left(\frac{1}{d_{n}}*D_{t}\right)-e^{\prime }+r_{t}(n-1)\to u_{s} \end{array}\right.\;$$ (5)

The hidden layer is derived from the above equation, and it is represented as $\;H_{\text{0}}$. The identification is done on an interval basis with the previous state, and it is given as the input for the first layer, where it acts as the current state $\;\left(v_{t}-u_{s}\right)-v_{n}$, if there is any loss or error occurs, then training is given to the particular signal, and it is labelled as $\;r_{t}$. This case illustrates the training section for both the current and previous states, providing a solution without any loss or error data. The hidden layer is used to hold the information of the last state, which reduces the complexity of the state process $\;v_{t}\left(d_{\text{0}}\right)\times s_{l}\to M_{e}$. The discussion about the current state and its evaluation using a hidden layer is derived mathematically in the following Equation (6),


$$u_{s}\left(C_{a}\right)=v_{t}\left(d_{\text{0}}\right)\to s_{l}\times \prod_{A\prime \in e\prime }L_{s}\left(D_{t}\right),\;for\;all\;v_{t}\;\forall \;S_{r}$$

$$u_{s}\left(C_{a}\right)=d_{n}+\prod_{v_{n}}\left(D_{t}(e^{\prime }-L_{s})\right)+\left(S_{r}+A_{e}\right)$$

$$u_{s}\left(C_{a}\right)=w_{g}\left(d_{\text{0}}\left(s_{l}\right)\right)\to D_{t}\left(L_{s}\right)\times \prod_{u_{c}\in B_{\text{0}}}d_{n}*r_{t}\left(s_{l}\right)-e\prime$$

$$u_{s}\left(C_{a}\right)\;=\sum_{d_{\text{0}}\in pa\;}^{u_{c}}\left\{{\left(D_{t}+d_{w}\right)}^{\text{2}}\right\}\times w_{g}\left(s_{l}\right)-v_{t}$$ (6)

**Fig 4:**
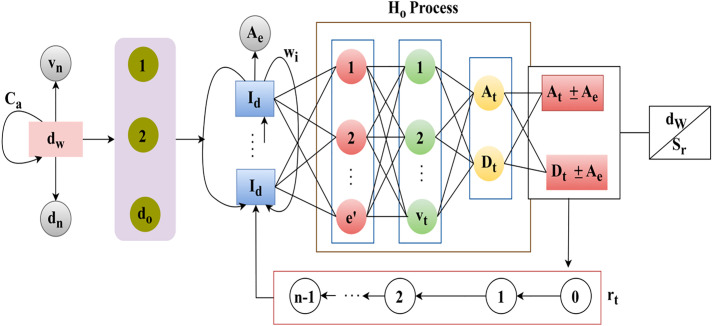
RL process for sampling rate fixation.

The calculation of the current state is used under the hidden layer, where it defines the analysis for the varying interval of time and executes the signal processing $\;\left(v_{n}-d_{n}\right)\times u_{c}$. This case defines the training part for the errors and losses during the communication $\;d_{w}\times \left(v_{t}-u_{c}\right)$. The RL process for fixing the sampling rate is presented in Fig 4.

The role of RL is to identify the $A_{t}$ and $D_{t}\forall S_{r}$ in any $d_{w}$ intervals. The first assignment is the $(C_{a},v_{n},\;and\;d_{n}\forall$ any $d_{w}$ such that $S_{l}$ segregates $\;d_{o}$. If the segregation is successful, then $\;I_{d}\forall \left(\text{1 }to\;A_{e}\right)$ the instance is validated for $w_{i}$ and $\;F_{a}$. This case is true for $\left(\frac{e^{\prime }-L_{s}}{\sum_{{(F}_{a}+w_{t})}B_{o}}\right)$ = high and for the low case e′ from ($v_{t}$ and $\;D_{t}$) occurrences are extracted. Depending on the $A_{t}$ or $D_{t}$ representation for $e^{\prime }=\text{0}$ achievement $S_{r}\forall d_{w}$ is decided. Thus, from the congruency of $\left(e^{\prime },v_{t},D_{t}\right)$ then $\left(A_{t}+A_{e}\right)$ and $\left(D_{t}\pm A_{e}\right)$ decides the $S_{r}$. In the training process, [o, tp and (n−1)] iterations are pursued to update $I_{d}\forall A_{e}$ such that $H_{o}$ is further organized. Here, $v_{t}$ is referred to before the $A_{t}$ (or) $D_{t}$ detection whereas $u_{s}$ defines the $\left(A_{t}\pm A_{e}\right)$ (or) $\left(D_{t}+A_{e}\right)$ for $S_{r}$ (Fig 4). Here, weights are assigned for every input in the hidden layer that detects the losses in the signal. The weights are used in the input, where the extraction is the output associated with reducing the output state. Thus, the current state uses the weigh function to address the losses during the communication. The training is given based on the input weight computed using the equation below (7).


$$\begin{array}{c} r_{t}\left(w_{g}\right)=G_{0}\times \left\|{(B}_{0}\times e^{\prime })-D_{t}\right\|+\sum_{L_{s}}^{e^{\prime }}\left(A_{e}+S_{r}\right)\times I_{d},\;for\;all\;I_{d}\approx u_{c} \end{array}$$

$$r_{t}\left(w_{g}\right)=d_{w}+\left(u_{c}-p_{a}\right)\times \frac{1}{d_{n}},\;where\;p_{a}\in v_{t}$$ (7)

$$r_{t}\left(w_{g}\right)=u_{c}+\left(A^{\prime }\times d_{n}\right)+\sum_{H_{0}\in s_{l}}\left(I_{d}+C_{a}\right)-v_{n},\;for\;all\;v_{n}\in G_{0}$$

**Fig 5:**
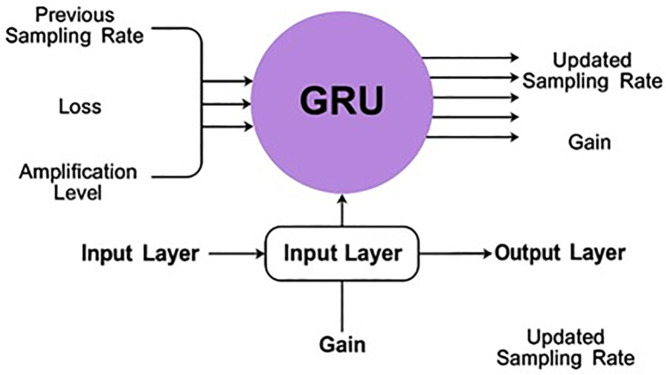
Process of recurrent learning.

The training is given based on the weight assigning process, where the assigning is defined as $\;G_{\text{0}}$, for the input signal to reduce the loss of communication. This represents the communication from the base station signal input. Here, the weights are assigned $\;G_{\text{0}}\left(w_{g}\right)+d_{n}\to D_{t}-u_{c}$, where the loss is detected $\;D_{t}(L_{s})$. This is processed with the sampling rate and amplification levels. If some losses or errors occur due to communication failure, a training session is given. This training is conducted using recurrent learning, where weight assignment is employed to address the losses. To modify the amplification and sampling parameters, as well as to segregate the temporal signal behavior, we apply a GRU-based recurrent architecture within the scope of the recurrent learning mechanism in the proposed DALFM module. For this purpose, a Gated Recurrent Unit (GRU) is suitable, as it performs better than LSTM and uses less power, making it suitable for UAV and 5G edge scenarios shown in Fig 5.

The GRU model takes as input a sequence of historical signal characteristics and provides optimized control signals during the upcoming downlink intervals. In particular, the model consists of an input layer, a hidden GRU layer with 64 units, and a dense output layer that outputs the updated values for the sampling rate and gain. The amplification level is analyzed at different time intervals and is expressed in Equation (8).


$$\begin{array}{c} A_{e}\left(v_{n},A^{\prime }\right)=B_{\text{0}}+\left(\frac{i_{\text{0}}-x_{m}}{D_{t}\times d_{w}}\right)\times \prod_{u_{c}\in r_{t}}^{d_{\text{0}}}\left(L_{s}-e^{\prime }\right)\times p_{a},\;where\;d_{w}\in S_{r} \end{array}$$

$$A_{e}\left(v_{n},A^{\prime }\right)=G_{\text{0}}\left(w_{g}\right)\times r_{t}+\left(d_{n}*B_{\text{0}}\right)+\frac{C_{a}+w_{g}}{\sum_{v_{t}-u_{s}}s_{l}},\;for\;all\;w_{g}\in r_{t}$$

$$A_{e}\left(v_{n},A^{\prime }\right)=\prod_{w_{i}}\left(q_{c}+s_{l}\right)+\frac{H_{\text{0}}}{d_{n}},\;for\;all\;d_{n}\in q_{c\;}$$ (8)

**Fig 6:**
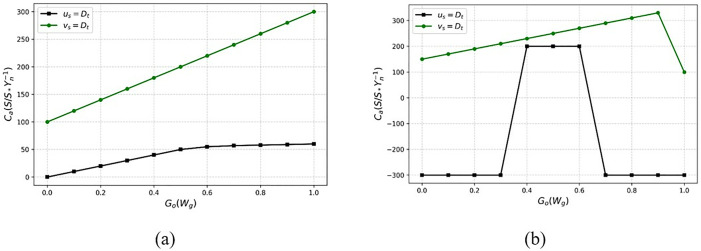
Amplification loss function rate analysis for weights assigned.

The analysis is carried out for the amplification level using a training phase that reduces communication losses. Here, the weight assignment is done to the amplification of the loss function, which is done on recurrent learning $\;w_{g}\left(G_{\text{0}}\right)+r_{t}(d_{n})$. The $\;G_{\text{0}}(w_{g})$ based $\;C_{a}$ rate is analyzed using Fig 5 using $\;v_{t}$ and $\;u_{s}$ variants for $\;A_{t}$ and $\;D_{t}$. The $C_{a}$ behaves in the $S_{r}$ allocation rate for $G_{o}\left(w_{g}\right)$ demanding $\left(\frac{S_{r}}{S_{r}}\times V_{n}^{-1}\right)$ in any $S_{l}\in d_{w}$. The $H_{o}$ process defines the congruency for maximum sampling across differentiable $w_{i}$ and $\;F_{a}$. Using the $(A_{t}\pm A_{e}$and $\;\left(D_{t}\pm A_{e}\right)$ for adaptable $S_{r}$ under $v_{t}$ and $u_{s}$ (vice versa), the $C_{a}$ is defined. The $r_{t}$ then decides the $G_{o}\left(w_{g}\right)$ (increase/decrease) from which the states of $D_{t}$ or $A_{t}$ are swapped. Therefore, normalized capacity adjustment defines the multiple states of the $\left(q_{c}+s_{l}\right)$ to maximize the sampling rate. For an even sampling without e′, the $C_{a}$ must satisfy the following:$\;L_{S}\left(D_{t}\right)\to \left(u_{c}-v_{n}\right),\;\left(S_{r}\times A_{e}\right)=\left(S_{r}*D_{t}\right)$ provided $\;\left(D_{t}-u_{c}\right)>0$ in any $\;v_{n}$. Therefore, these different requirements leverage the need for $G_{o}\left(w_{g}\right)$ assignment balancing the above conditions given in Fig 6. Post the $\;C_{a}$ analysis, the gain estimation is defined in the following sub-section.

### 3.4. Gain estimation

Based on the loss, the maximum and minimum transmission gains are estimated for which the amplification is pursued. This gain estimation is validated using recurrent learning by augmenting the least and maximum losses observed in different downlink intervals. In computing, this time interval varies for the amplification levels and finds the maximum and minimum range, which is symbolized as $\;x_{m}\;and\;i_{\text{0}}$. The loss detection is computed in the following Equation (9).


$$I_{d}\left(L_{s}\right)=p_{a}\left(D_{t}\right)+\left(S_{r}+A_{e}\right)\times \sum_{M_{e}\in u_{c}}^{w_{g}}e^{\prime }+d_{n},\;where\;e\prime \in u_{c}$$

$$I_{d}\left(L_{s}\right)=\frac{\text{1}}{d_{n}}+q_{c}\left(s_{l}\right)\times d_{w}+\sum_{A^{\prime }}\left(v_{t}-u_{s}\right)-v_{n}$$

$$I_{d}\left(L_{s}\right)=\frac{d_{\text{0}}}{A\prime }+D_{t}\left(e^{\prime }\right)+C_{a}\left(w_{g}\right)+\sum_{M_{e}}{(r}_{t}+d_{\text{0}})$$

$$I_{d}\left(L_{s}\right)=\frac{C_{a}}{w_{g}}-e^{\prime }+\left(G_{\text{0}}-i_{\text{0}}\right)+\sum_{u_{s}}q_{c}-u_{s}$$ (9)

The above equation identifies the loss that occurs when communication leads to signal processing. Executing this mechanism illustrates the weight-based training section. This approach is implemented in recurrent learning, where the previous state is used to compute the transmission gain process using Equation (10) below.


$$V_{\text{0}}=\left(s_{l}+q_{c}\right)\to d_{w}\times \prod_{r_{t}}\left(w_{g}+d_{\text{0}}\right)\times G_{\text{0}}\left(d_{\text{0}}\right)\times A_{e}+S_{r}$$

$$w_{g}\left(d_{\text{0}}\right)=\left(q_{c}+s_{l}\right)\times \left(e^{\prime }-L_{s}\right),\;where\;L_{s}\;\in r_{t}$$ (10)

**Fig 7:**
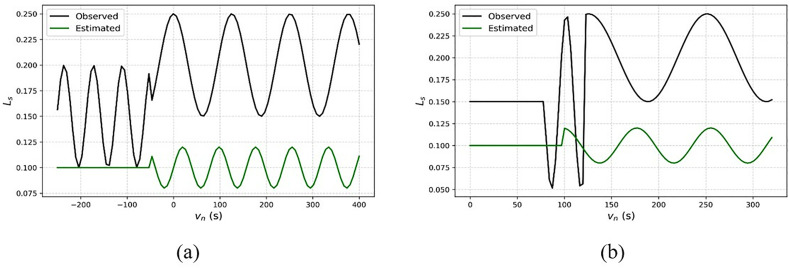
${𝐋}_{S}$ estimated and observed (a) amplification loss function and (b) time interval.

The verification is followed up, and it is described as $\;V_{\text{0}}$, where it indicates the training part with the weight-assigning factor. This illustrates $\;D_{t}\left(L_{s}-e^{\prime }\right)\times v_{n}$, where it defines the assigning factor for the transmission gain. The observed and anticipated path loss are shown in Fig 7. The output estimate is derived from a reference value using a weighted combination of factors and the system’s delay characteristics. The extent to which route losses are expected to occur depends on the current health of the communication network. This variation might be influenced by system-specific factors such as transmission rate and amplification level. Depending on the actual signal-to-noise ratio or amplification settings, the reported path loss might change in response to different communication scenarios. As a result, amplification levels and noise effects alter the difference between the transmitted and received signals, thereby decreasing route loss before allocating resources. As the capacity factor grows, the difference between the broadcast and received signal levels varies, allowing for a more precise assessment of route loss. Here, when capacity increases, the amplification level determines the updated value of the delivered signal. It is possible to reduce route loss effectively by adjusting the transmission settings. Keeping the sample rate and amplification in check as the downlink forward signal processing continues guarantees proper signal quality. A communication system’s ability to withstand changes in the network’s status and the external environment is crucial to its success.

The hidden layer trains the error-based path and resolves the efficient communication for the air-to-terrestrial module. After this, the decision is made using recurrent learning to improve the transmission gain, formulated in Equation (11) below.


$$s_{d}=\left(v_{t}-u_{s}\right)\times \prod_{u_{c}\in A_{e}}^{S_{r}}s_{l}\to H_{\text{0}}\times \frac{\text{1}}{d_{n}}+\left(w_{g}(G_{\text{0}})\right)$$

$$s_{d}=q_{c}\left(s_{l}\right)\times \prod_{C_{a}}\left(I_{d}+d_{\text{0}}\right)\times u_{c}-v_{n},\;for\;all\;I_{d}\;\in S_{r}$$

$$\begin{array}{c} s_{d}=\left(d_{w}-p_{a}\right)\times \prod_{D_{t}}\left(L_{s}-e^{\prime }\right)+M_{e}-A^{\prime }+t_{g} \end{array}$$ (11)

**Fig 8:**
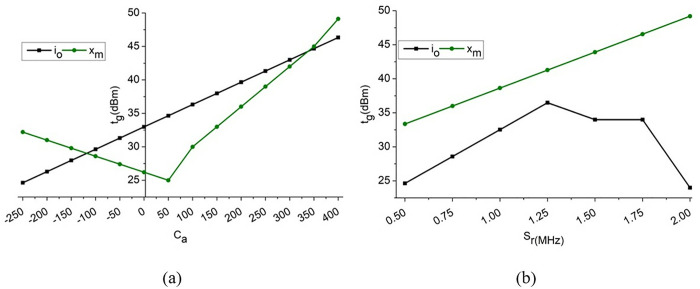
Transmission gain for (a) amplification loss function and (b) sampling rate.

The decision is $\;s_{d}$, whereas transmission gain is represented as $\;t_{g}$ where it is improved by reducing the amplification loss and increasing the sampling rate. This is performed based on the analysis step $\;A^{\prime }\left(d_{\text{0}}\times q_{c}\right)-v_{n}$. Based on this execution step, the identification of data is done with the recurrent learning concept $\;u_{c}\left(A_{e}+S_{r}\right)\times d_{\text{0}}$. Upon processing this state, the decision is made to enhance the transmission gain with maximum range, which is achieved by developing a recurrent learning methodology. This module identifies the system gain during frequency sampling at different amplification levels. The amplification is required to prevent transmission losses between distinct intervals across various pervasive downlinks. The $\;t_{g}$ for different $\;C_{a}$ and $\;S_{r}$ is analyzed graphically and presented in Fig 8. Fig 8(a) shows variation in transmission gain versus amplification loss and sampling rate. The new DALFM dynamically adjusts amplification levels in response to real-time signal conditions, thereby minimizing path loss and maximizing transmission efficiency. The results show that DALFM always enhances transmission gain over conventional methods by adaptively reacting to changing downlink intervals. This performance demonstrates its effectiveness in preventing signal degradation and ensuring stable communication in 5G air-to-terrestrial networks. In Fig 8(b), the $t_{g}$ for $\;C_{a}$ and $S_{r}$ are analyzed and graphically illustrated. The proposed module allocates optimal $C_{a}$ based on $L_{s}$ detection under $v_{t}$ and $\;u_{s}$. This requires $\left(A_{t}\pm A_{e}\right)$ (or) $\begin{array}{c} \left(D_{t}\pm A_{e}\right)\forall \;S_{r} \end{array}$ under $\;H_{o}$ outputs. Therefore $W_{g}\left(d_{o}\right)$ are substituted to balance $\left(L_{s}-e^{\prime }\right),\;\left(\frac{i_{o}-x_{m}}{D_{t}*d_{w}}\right)$ for $d_{n}\to D_{t}-u_{c}$ and $\begin{array}{c} r_{t}\left(W_{g}\right)\forall \;I_{d}≃u_{c} \end{array}$ and $\;W_{g}\left(s_{l}\right)-e^{\prime }\left(L_{s}\right)=\left(r_{t}+A_{e}\right)$. Thus $\left(v_{n}-d_{n}\right)\times u_{c}$ infers the $d_{w}\times \left(v_{t}-v_{c}\right)$ for various iterations to retain a high $t_{g}$ as presented. 5G air-to-terrestrial communication neural network methods’ accuracy via a downlink loss function tailored to handle interference, channel fading, and signal deterioration. This customized strategy is anticipated to enhance Important parameters, such as packet error rates, signal-to-noise ratio (SNR), and overall transmission reliability. When optimizing communication goals, DALFM shows better accuracy than standard neural network techniques that employ general-purpose loss functions. The computational complexity also increases, as the amplified loss function may require more sophisticated computations and additional resources for real-time training and inference in dynamic network environments, which is the cost users incur to obtain this advantage.

DALFM necessitates periodic learning in dynamically varying amplification (A) and sampling rate (S to support ongoing downlink communication. Unlike other conventional techniques that use fixed transmission parameters, DALFM updates dynamically based on historical measurements of path loss (PL) and current instantaneous signal variations. DALFM monitors each time interval t, the previous amplification state $A_{t-1}$ and transmission gain $G_{t-1}$, using them to predict the optimal values for the next interval. The recurrent learning model identifies trends in path loss (${PL}_{t}$) and Doppler shifts $D_{t}$, dynamically adjusting the signal gain $G_{t}$ to counteract degradation. The update equation governs this process using $A_{t}=A_{t-1}+\alpha \left(PL_{t}-PL_{t-1}\right)-\beta (D_{t}-D_{t-1})$ where α and β are learning coefficients that regulate the adaptation rate according to path loss and Doppler variation.

The model continually improves its prediction by adjusting its learning weights based on both previous and current transmission conditions. It employs amplification and sampling adjustments in response to instantaneous variations, thereby avoiding signal distortion. In addition, the sampling rate $S_{t}\;$is dynamically chosen according to the transmission error $E_{t}$: as $S_{t}=S_{t-1}+\gamma (E_{t}-E_{t-1})$, where γ is a weight parameter providing the optimal balance between error correction and transmission stability. By incorporating recurrent learning, DALFM introduces an adaptive self-optimization function, enabling a high-level ability to enhance signal stability, reduce transmission errors, and maintain uninterrupted communication quality. Therefore, it is a well-matched solution for 5G air-to-terrestrial networks, especially in high-mobility conditions where path loss, Doppler shift, and channel changes pose extremely formidable challenges.

## 4. Results and discussion

### 4.1. Experimental setup

The proposed module is experimentally tested using an open network scenario with 5 air vehicles (UAVs) communicating with 3 base stations, covering a 500m communication range. The UAV’s altitude is 5 km above ground level, with a maximum transmit power of 2 W and an operating frequency of 2.4 GHz. The BS transmit power is set as 40dBm maximum, and the $\;d_{w}$ The communication frequency is 2 GHz operating at 10 G cycles/second. The maximum sharing interval is set at 320 seconds, the maximum achievable gain is 60 dB, and the amplification is set at a rate of 4 dB. The lowest bandwidth allocation is 4.5 MHz, and the maximum is 7 MHz. Additionally, the experimental setup features 200 users and micro stations. Simulation was performed using MATLAB and NS-3 for network-level analysis and signal processing. The 5 km altitude of the UAV was utilized to reduce interference and ensure effective signal coverage through transmit power (2W for UAV and 40 dBm for BS). The operating frequencies of 2.4 GHz and 2 GHz are compliant with 5G and IoT use cases. Stability in communication was confirmed with a sharing interval of 320 seconds, and a bandwidth of 4.5–7 MHz was utilized to ensure compliance with the 5G network requirements. These parameters provided a real-world test environment, enabling DALFM to realistically counteract path loss, maximize signal gain, and dynamically vary amplification in high-mobility 5G air-to-terrestrial communication networks. The DALFM framework employs a recurrent learning model—specifically, a gated recurrent unit (GRU)—to dynamically adjust the downlink amplification and sampling rates of the unit as they evolve due to degradation. Training takes place offline and utilizes a synthetic dataset created under various mobility, interference, and path loss scenarios, enabling the model to capture temporal dependencies in the signals. The GRU is then integrated into the signal processing pipeline, where it adjusts gain control and sampling in real-time based on feedback from the system. This streamlined form enables the deployment of the model on UAV communication modules or 5G edge devices, which have limited processing capabilities. According to the discussion, the parameters utilized in this work are tabulated in Table 2.

**Table 2:** Parameter settings.

Parameter	Value	Parameter	Value
Number of UAVs	5	Amplification Rate	4 dB
Number of Base Stations	3	Max Achievable Gain	60 dB
UAV Altitude	5 km	Bandwidth Allocation	4.5 MHz – 7 MHz
Communication Range	500 m	Maximum Sharing Interval	320 seconds
UAV Transmit Power	2 W	Number of Users	200
BS Transmit Power	40 dBm	Simulation Tools	MATLAB, NS-3
UAV Operating Frequency	2.4 GHz	Path Loss Models Used	Rayleigh and Rician fading
Downlink Frequency (BS to UAV)	2.0 GHz	Recurrent Learning Model	Gated Recurrent Unit (GRU)
Sampling Frequency	10 G cycles/second	GRU Training Mode	Offline (synthetic signal datasets)
		Deployment Target	UAV communication module/ 5G edge

### 4.2. Assessment with metrics and selected methods

**Fig 9:**
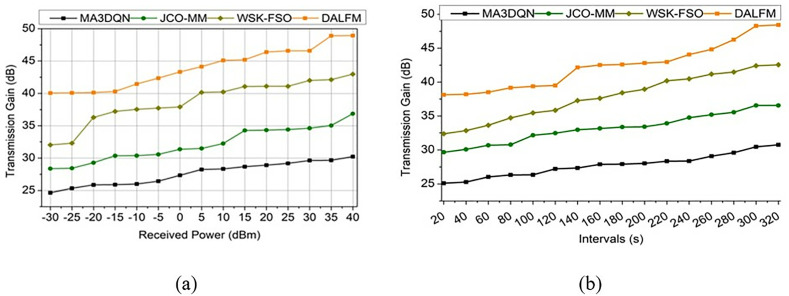
$\;t_{g}$ Performance assessment of transmission gain (a) received power and (b) intervals.

Using the above experimental setup, transmission gain, path loss, sampling rate, error rate, and communication rate metrics are assessed. The assessment is presented as a comparative derivation with the MA3DQN [20], JCO-MM [35], and WSK-FSO [28] methods, as referenced in the related works section. These metrics are studied under received power (−30 dBm to 40 dBm) and interval (20 s to 320 s) variants. The transmission gain for the proposed work is high for varying received power and intervals. The scope here is to improve the transmission gain that is associated with sampling rate and amplification levels that lead to maximum and minimum range $\;\left(S_{r}+A_{e}\right)-t_{g}$. This case illustrates the downlink communication and reduces the losses and errors in signal sharing. It is performed as the transmission gain approach, $t_{g}=\sum_{w_{i}}^{F_{a}}\frac{\left(A_{e}+S_{r}\right)*s_{l}-v_{n}}{B_{\text{0}}-(A\prime {)}^{\text{2}}}+\left(p_{a}*D_{t}\right)$. The transmission gain performance between the existing methods and the proposed module is illustrated in Fig 9.

**Fig 10:**
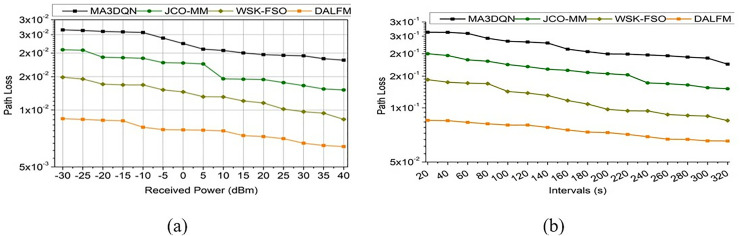
Performance assessment of path loss for (a) received power, (b) intervals.

This process illustrates the transmission gain that estimates the sampling rate and amplification levels that are associated with the different time intervals $\;v_{n}-t_{g}(p_{a}\times D_{t})$. Here, $\frac{L_{s}}{\prod_{u_{c}}D_{t}}+\left\{\left[\left(d_{\text{0}}+s_{l}\right)\times \left(d_{w}-u_{k}\right)\right]-v_{n}\right\}$ is performed for signal-based data processing. The path is defined as $\;p_{a}$ that illustrates $\;p_{a}>t_{g}-u_{c},\;where\;u_{c}\in L_{s}$. The communication leads to a transmission gain estimated as ${\;d}_{\text{0}}\left(u_{c}\right)$, this process $\;\left(d_{w}-u_{k}\right)\approx \left(S_{r}-A_{e}\right)$. This executes the transmission gain, $\;\left(t_{g}*p_{a}\right)+\sum_{r_{t}}\left(w_{i}+F_{a}\right)\times u_{c},\;where{\;r}_{t}\in w_{g}$ (Fig 10). The path loss decreases by addressing the path losses where the error is sorted out as $\;\left(L_{s}-e\prime \right)*D_{t}$. This evaluation step indicates $\;p_{a}\left(D_{t}\right)*\left(\frac{s_{l}+d_{\text{0}}}{\sum_{A_{e}}S_{r}}\right)u_{c}+\sum_{d_{w}}^{u_{k}}\left(u_{c}-p_{a}\right),\;for\;all\;p_{a}\in v_{n}$. Based on this evaluation, the performance assessed for path loss is illustrated in Fig 10. The suggested DALFM offers apparent benefits for practical use due to its performance, which considerably enhances critical communication characteristics. The technology is ideal for high-mobility settings, such as UAV communications, because it allows for the dynamic adjustment of the sample rate, resulting in a 10.67% improvement in communication efficiency. Important for intelligent transportation systems and driverless vehicles, this guarantees more dependable data transfer. The loss estimation enhances the communication between the air-to-terrestrial modules and identifies optimal $\;d_{w}\left(u_{c}*d_{n}\right)-v_{n}$. This states the sampling rate and amplification levels, and improves the transmission gain $\;t_{g}\left(S_{r}+A_{e}\right)\times \frac{B_{\text{0}}}{i_{\text{0}}-x_{m}}$, it is expressed as, $I_{d}=\frac{\text{1}}{d_{n}}+\left\{\sum_{p_{a}\in u_{c}}^{q_{c}}s_{l}+\left(d_{w}\times D_{t}\right),\;for\;all\;d_{w}\;\forall \;A_{e}\right\}\times \;\left[\sum_{u_{k}}^{d_{w}}\frac{C_{a}}{p_{a}}\times \sum_{q_{c}\in v_{n}}^{s_{l}}M_{e}+\left(S_{r}+A_{e}\right),\;all\;S_{r},A_{e}\in d_{w}\right]\times d_{w}\times \frac{M_{e}}{\sum_{C_{a}}A^{\prime }+u_{c}}$. It exhibits the amplified loss function that illustrates the sampling rate on different time intervals $\;d_{w}(S_{r}-v_{n})$. This processing step is defined as $I_{d}\left(v_{n}(n)-v_{t}\right)=\left(A^{\prime }+S_{r}\right)+d_{n-\text{1}}\times \prod_{d_{w}\in u_{c}}^{A_{e}}s_{l(n-\text{1})}+\left(\frac{\text{1}}{d_{n}}\times D_{t}\right)-e^{\prime }+r_{t}(n-\text{1})\to u_{s}$.

**Fig 11:**
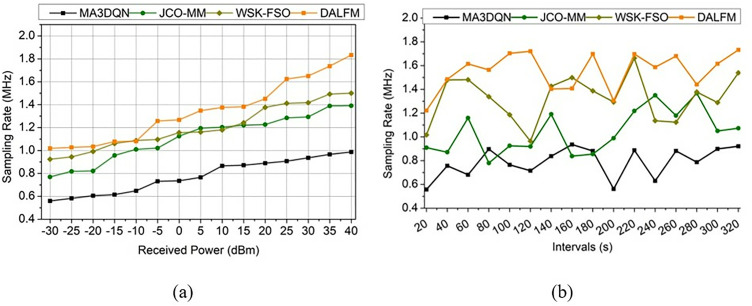
Sampling rate comparative illustration between existing methods and proposed module; (a) received power, (b) intervals.

It is used to evaluate the analysis method for sampling rate and exhibits the detection of the signal by reducing the loss and error $\;A^{\prime }\left(e^{\prime }-L_{s}\right),\;for\;all\;A^{\prime }\approx D_{t}$. It defines the transmission gain $\;t_{g}\times \frac{\text{1}}{d_{n}\left(s_{l}\right)}\times I_{d}$ (Fig 11). The sampling rate for the proposed work defines the higher value range with the processing of received power and intervals $\;\left(v_{n}*S_{r}\right)>w_{g}(G_{\text{0}})$. The 8.12% boost in transmission gain further demonstrates DALFM’s capability to maintain a constant signal strength across a wide range of distances and in complex environments. The effects of environmental changes and physical barriers, such as buildings and weather, on the transmission quality of 5G air-to-terrestrial communication networks, which include aircraft and drones, are substantial. Using the above derivative, the $\;S_{r}$ assessment as a comparative illustration is given in Fig 11. From this derivative, the sampling rate is defined and improved, which indicates a higher transmission gain. In this work, weights are assigned to reduce loss and error rates. The processing is carried out as, $S_{r}$.Whereas it is computed as, $D_{t}\left(S_{r}\right)=\frac{C_{a}}{w_{g}}-e^{\prime }+\left(G_{\text{0}}-i_{\text{0}}\right)+\sum_{u_{s}}q_{c}-u_{s}$. The functionality is used to evaluate the sampling rate as, $\frac{\text{1}}{d_{n}}+q_{c}\left(s_{l}\right)\times d_{w}+\sum_{A^{\prime }}\left(v_{t}-u_{s}\right)-v_{n}$, thus, it shows a higher sampling rate (Fig 11).

**Fig 12:**
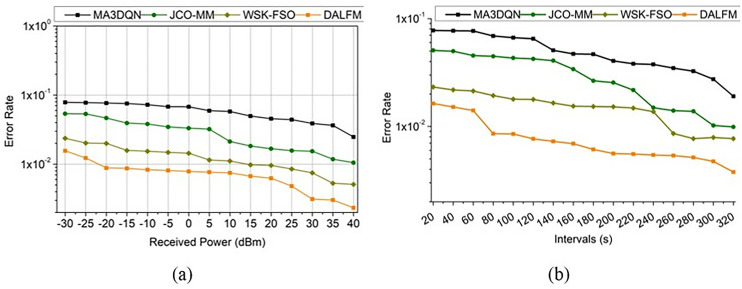
Error rate analysis for (a) received power and (b) intervals.

The error rate for the proposed work decreases, and it is associated with the received power and the interval $\;e^{\prime }+D_{t}\left(w_{i}+F_{a}\right)\times \frac{\sum_{s_{d}}^{A\prime }\left(v_{t}-u_{s}\right)}{H_{\text{0}}+G_{\text{0}}}$ and this is performed under the concept of recurrent learning, which assigns weights for the computation. The error rate is comparatively analyzed for the different received power and intervals, as shown in Fig 12.

**Fig 13:**
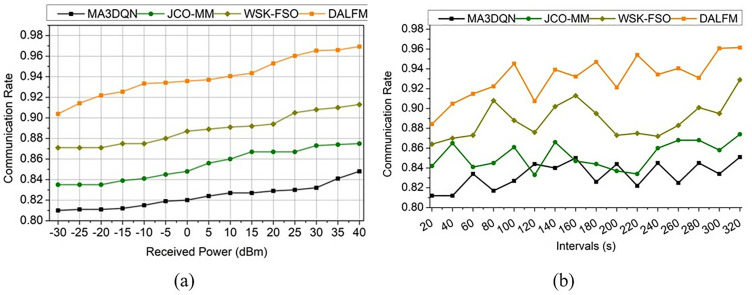
Communication rate assessment (a) received power (b) intervals.

The evaluation step is considered for the varying time interval and exhibits the detection of the error rate $\;A^{\prime }\left(A_{e}-S_{r}\right)\times \prod_{u_{c}}\left(e^{\prime }-D_{t}\right)-v_{n},\;where\;D_{t}\;\approx r_{t}$ and this leads to addressing the losses and errors in communication $\;u_{c}+D_{t}\left(e^{\prime }-L_{s}\right)\times \sum_{q_{c}}^{d_{\text{0}}}\left(I_{d}\times A_{e}\right)-v_{n}$ and it is expressed as, $e^{\prime }\left(D_{t}\right)=\sum_{d_{w}}^{u_{k}}G_{\text{0}}\left(w_{g}\right)\times H_{\text{0}}\times \left\{\left(\frac{d_{\text{0}}\to u_{c}}{\sum_{q_{c}}s_{l}}\right)-p_{a}\right\},\;for\;all\;G_{\text{0}}\in w_{g}.$ The above equation defines the detection of the error rate that is associated with the weights assigned to the input signal $\;w_{g}(G_{\text{0}}+d_{\text{0}}\left(s_{l}\right))$. The prompt communication rate addressing the path loss is evaluated based on the error rate estimation. The representation for a comparative assessment is given in Fig 13. The communication rate increases for varying time intervals that are associated with downlink and uplink, where the loss and error rate are detected $\;L_{s}-e^{\prime }\left(D_{t}\right)-v_{n}*A^{\prime },\;where\;A^{\prime }\in p_{a}$. This analysis calculates path losses and provides the transmission gain, indicating that path losses are detected for each time interval, taking into account fading and shadowing. To mitigate these problems, weights are assigned, which show a higher communication rate for varying received power and interval by addressing the path loss and error. Traditional communication methods struggle to sustain connections in rural or disaster-stricken areas, but DALFM’s 9.57% reduction in route loss suggests that this technology may improve service quality in these locations. Internet of Things (IoT) devices in remote areas and emergency networks are supported by this route loss mitigation, ensuring service continuity during critical moments. By efficiently adjusting to changing conditions in real-time, the suggested DALFM improves network stability and reduces errors, making it superior to alternative solutions. For example, resource allocation methods like MA3DQN may not always be effective in situations with high movement or disturbance. However, DALFM’s dynamic sampling and amplification guarantee stable network performance even in highly dynamic circumstances, allowing for high-quality communication. From the above performance assessment discussion, the following highlights are provided: For the received power of 40 dBm, the proposed module is found to achieve 8.34% high $\;t_{g}$, 9.83% high $\;S_{r}$, and a 9.06% high communication rate. Besides, this module reduced $\;L_{s}$ by 10.4% and  e′ by 11.13%. For the $\;v_{n}$ = 320s, the proposed module is found to achieve an 8.12% high $\;t_{g}$, 10.67% high $\;S_{r}$, and a 9.68% high communication rate. Besides, this module reduced $\;L_{s}$ by 9.57% and  e′ by 11.43%.

According to the comprehensive results, the suggested Down-Link Amplified Loss Function Module (DALFM) outperforms MA3DQN, JCO-MM, and WSK-FSO in necessary performance measures, including sampling rate, route loss reduction, and transmission gain. For example, regarding dynamic intervals, DALFM improves the sample rate by 10.67% and the transmission gain by 8.12%. The model’s recurrently-guided adaptive amplification and sampling approach significantly contributes to its ability to maintain reliable communication in dynamic environments. While MA3DQN is great at allocating resources, it has poor sampling rates in high-mobility situations since it doesn’t consider environmental changes in real-time. The WSK-FSO method is similar in that it reduces turbulence in FSO networks; however, it is limited to line-of-sight networks and doesn’t address transmission loss in ADS-B networks. However, the suggested model’s adaptive gain estimate method shows better dependability on various situations, cutting route loss by 9.57 percent.

Another noteworthy development is that DALFM’s error rate is lower than that of JCO-MM. The recurrent learning module is responsible for this enhancement because it utilizes past state data to adjust signal amplification dynamically. In contrast to JCO-MM, which has a higher error rate because it can’t adapt as well to downlink transmission, DALFM ensures low latency and good reliability by properly balancing the sampling rate and amplification. The suggested method has substantial practical uses in optimizing communication networks across various sectors. Enhanced transmission gain and excellent sample rates enable effective and dependable data transfer in 5G air-to-terrestrial communication, particularly in dynamic environments. Intelligent transportation systems benefit significantly because they improve the dependability of Vehicle-to-Everything (V2X) communication, which supports autonomous vehicle platooning and real-time traffic management by reducing path loss. Internet of Things (IoT) applications, such as smart grids and industrial automation, rely on accurate data being collected and transmitted due to increasing sample rates. Another critical aspect of aerial networks is the need for enhanced signal dependability and decreased degradation. This is especially true for UAVs in disaster response, surveillance, and delivery services.

Table 2 summarizes the advantages of DALFM over existing methods in extrapolating their limitations to dynamic environments. MA3DQN cannot dynamically adapt in real time, thus offering inferior resource allocation, while WSK-FSO is only functional in line-of-sight scenarios. DALFM surpasses them by implementing real-time transmission parameter adaptations, which optimize and record a 10.67% increase in sampling rate efficiency and a 9.57% reduction in path loss (Table 3).

**Table 3:** Comparison of DALFM with existing methods in dynamic 5G air-to-terrestrial network.

Method	Key Focus	Limitations in Dynamic Environments	DALFM Advantage Over Existing Models	Quantitative Improvement
MA3DQN	User association and resource allocation	Lacks dynamic sampling adjustments, struggles with real-time adaptation, leading to higher latency	DALFM dynamically adjusts sampling and amplification levels for real-time optimization	10.67% higher sampling rate efficiency
WSK-FSO	Free-space optical (FSO) communication with turbulence mitigation	Limited to line-of-sight networks, cannot compensate for Doppler shifts or non-line-of-sight conditions	DALFM adjusts transmission parameters based on real-time conditions, improving adaptability	9.57% reduction in path loss
DALFM	Recurrent learning-based transmission optimization	–	Continuously adapts to varying network conditions, ensuring robust downlink communication.	Enhanced adaptability in dynamic 5G air-to-terrestrial networks

While sustaining solutions to spectrum management, mobility modeling, and atmospheric compensation with ML-based compensation models MA3DQN, JCO-MM, and WSK-FSO, respectively, the novelty of DALFM centers on dual-layer adaptive optimization with amplification gain tuning and dynamic downlink sampling synchronization via recurrent learning. Unlike previous models, which consider path loss and Doppler effects in a vacuum or static environments, DALFM is tailored for high-mobility air-to-terrestrial 5G communication, where signal propagation rapidly fluctuates. The core innovation is the feedback mechanism, which adapts contextually based on real-time degradation signals through amplification and sampling. The integrated approach absent in the mentioned models addresses aerial dynamics, which remains a crucial yet under-researched concern for 5G air-to-ground networks. The experimental results, which provide an 8.12% gain in transmission strength, a 10.67% improvement in sample rate alignment, and a 9.57% reduction in path loss, validate the proposed approach’s further distinction from conventional incremental enhancements that claim to contribute to advancing the technology.

The validation of this study through simulations in MATLAB and NS-3 is grounded in actual communication parameters, ensuring relevance to practical hardware frameworks. The experiment utilizes standard UAV operating altitudes (5 km), practical transmit powers (2 W for UAVs and 40 dBm for base stations), as well as 2.4 GHz and 2 GHz frequencies, which fall within the 5G and IoT communication bands. Also considered are dynamic bandwidth allocation of 4.5 to 7 MHz, realistic path loss models that include Rayleigh and Rician fading, and user density ranging from 200 users with micro base stations to reflect urban and aerial communication environments. Although physical hardware implementation was not part of this phase, the modular and adaptable design of the DALFM module enables future integration with SDR (Software Defined Radio) frameworks or embedded 5G chipsets for backfitted field deployments. This path from simulation to hardware illustrates how the proposed approach can be actualized in practice and underscores its validation potential through subsequent testbed or prototype air-to-terrestrial communication network deployments.

### 4.3. Discussion on real-world deployment challenges

Although the results prove the ability of DALFM to achieve optimal transmission gain and minimize path loss, actual implementation in real life has some problems that need to be addressed:

- Computational Overhead: DALFM’s recurrent learning architecture also involves the continuous processing of downlink signal parameters, which can introduce computational complexity. In constrained systems, such as those in UAV-based networks, real-time deployment may be made unavoidable by optimization strategies, like model compression or the incorporation of edge computing, to curtail latency.
- Scalability to Large, Complex Networks: DALFM has been tested in a controlled 5G air-to-terrestrial network scenario. However, its behavior in highly heterogeneous environments, such as ultra-dense urban deployments or massive-scale satellite-terrestrial networks, requires further investigation. Future research will explore adaptive parameter adjustment and distributed learning techniques to scale up the approach.
- Integration with Existing 5G Infrastructure: Incorporating DALFM into current 5G topologies may necessitate modifications to network protocols and resource allocation rules. Integrating DALFM with existing infrastructure, particularly in dynamic multi-access technology environments, presents a significant challenge for practical deployment.

Future work will focus on addressing these issues to advance DALFM sufficiently for large-scale, real-time implementations with minimal computational overhead.

## 5. Conclusion

This article proposes and briefly describes the downlink amplified loss module for improving 5G-based air-to-terrestrial communication. The proposed module is reliable for streamlining 5G downlink communication that experiences high path loss due to invariable channel allocations. This module handled the above problem through adaptable sampling rates and amplification level decisions. In this process, the transmission gain is estimated and classified as high or low, for which amplification is required. The condition that the rate of amplification must nullify the path loss is verified using recurrent learning, which utilizes the current and previous states of the amplification levels. The linear optimization of the learning process allocates defined sampling rates individually for low and high transmission gain intervals. The communication intervals are assigned based on the learning decisions, and appropriate sampling rates are fixed. Therefore, the possibility of maximum interval allocation is high, which ensures an 8.34% transmission gain, a 9.83% high sampling rate, and 10.4% less path loss compared to existing models. Future work will focus on nonlinear optimization for channel allocation and sampling based on resource constraints. The constraints, such as time and utilization, will be addressed as a non-linear optimization problem to make the sampling rate adaptable.

Future work will concentrate on non-linear optimization methods for adaptive channel allocation and dynamic sampling to facilitate resource-efficient utilization. Real-time deployment models based on edge computing and low-latency processing will also be explored to enhance the practicality of DALFM in large-scale 5G and 6G networks.

### Symbols

**Table:** 

Variable	Description
$u_{s}(C_{a})$	The transmission performance parameter related to capacity.
$v_{t}(d_{0})$	The transmission performance parameter related to capacity.
$s_{l}$	Loss factor or scaling parameter applied during transmission.
$L_{s}(D_{t})$	Link scaling factor based on transmission delay, $D_{t}$ (Loss factor related to data rate).
$\begin{array}{c} A^{\prime } \end{array}$	Auxiliary or fixed parameter, potentially indicating additional environmental or configuration aspects.
$\begin{array}{c} e^{\prime } \end{array}$	Set representing environmental parameters or system configuration terms (e.g., interference).
$d_{w}$	Weighting factor associated with distance or environment in signal transmission.
$D_{t}$	The transmission data rate or throughput associated with the system’s communication capacity.
$r_{t}(s_{l})$	Function representing signal reliability over varying distance or environment sls_lsl.
$s_{r}$	The signal-to-noise ratio at the receiver node is affected by various transmission and environmental factors.
$A_{e}$	Amplification or attenuation at the receiver node, often based on signal degradation factors.
$p_{a}$	Probability associated with the path loss or attenuation factor in the communication model.
$w_{g}(d_{0}(s_{l}))$	The weighting function is related to the propagation distance and its associated environmental factors.
∏	Summation or product operator, typically used for representing aggregate systems or models.
$q_{c}$	Variable for the set of possible channel conditions or frequencies associated with transmission.
$u_{c}$	Communication nodes or channels are involved in the system’s signal exchange.
$M_{e}$	Environmental model parameter, likely referring to attenuation or ecological impact on signal strength.
$B_{0}$	A subset of user nodes or communication pathways for specific channel adjustments.
$p_{a}$	Power allocation is often used in resource distribution within a system.
ρ	The overall set of potential paths or configurations for transmission.
x(t)	Input signal as a function of time (continuous-time signal).
x[n]	Discrete-time input signal (sampled at intervals).
h(t)	Impulse response of the system (continuous time).
h[n]	Discrete-time impulse response of the system.
y(t)	Output signal of a system (continuous time).
y[n]	Discrete-time output signal of a system.
$C_{a}$	Communication capacity or a system’s total potential for carrying data (e.g., throughput or channel capacity).
$S_{r}$	Signal-to-Noise Ratio (SNR) indicates the desired signal level compared to the background noise.
ϕ	Phase shift is commonly used in systems with modulation and demodulation.
$\sigma^{2}$	The variance of noise typically represents the power of the noise in the system.
ν(t)	Frequency noise or offset in communication systems due to the Doppler effects.
p(t)	The probability density function is often used in modeling channel noise or errors.
$N_{0}$	Noise power spectral density is the power per unit bandwidth associated with thermal or background noise.
$W_{g}$	Weighting factor (e.g., for channel conditions, filters, or amplification factors).
R	Bit rate or symbol rate in communication systems.
T	Symbol period is the time duration of one symbol in digital communication systems.
$P_{tx}$	Transmission power is the power used by the transmitter to send signals.
$G_{t}$ and $G_{r}$	Gain of the transmitting and receiving antenna.
r	Distance between transmitting and receiving nodes (used in path loss calculations).
λ	Wavelength of the transmission signal.
θ	Angle of arrival or departure, often used in beamforming and antenna models.
